# Herpes simplex virus type 2 trends in relation to the HIV epidemic in northern Malawi

**DOI:** 10.1136/sti.2008.030056

**Published:** 2008-06-04

**Authors:** J R Glynn, A C Crampin, B M M Ngwira, R Ndhlovu, O Mwanyongo, P E M Fine

**Affiliations:** 1 Infectious Disease Epidemiology Unit, London School of Hygiene and Tropical Medicine, London, UK; 2 Karonga Prevention Study, PO Box 46, Chilumba, Malawi

## Abstract

**Objectives:**

It is unclear whether the high prevalence of herpes simplex virus type 2 (HSV-2) found in much of Africa predates the HIV epidemic or is, to some extent, a consequence of it. HSV-2 prevalence trends in a rural African community were assessed over a period in which HIV prevalence rose sharply, and antenatal clinic (ANC) surveillance was explored as a method of estimating community HSV-2 prevalence.

**Methods:**

HSV-2 seroprevalence was determined among community controls seen for case–control studies of mycobacterial disease in Karonga district, Malawi, in 1988–90, 1998–2001 and 2002–5, and in women attending ANC as part of surveillance for HIV in 1999–2000. Over this period HIV prevalence rose from 4% to 12%.

**Results:**

HSV-2 prevalence in all periods increased sharply with age and was higher in women than in men. After excluding migrants, there was no evidence of change in HSV-2 prevalence in the different periods. Women in the ANC group had lower HSV-2 prevalence than those in the community, but the ANC prevalence was a good approximation to the combined male and female prevalence for the same age group.

**Conclusions:**

This study suggests that HSV-2 was already widespread before the HIV epidemic and has not been greatly influenced by it. It also demonstrates that ANC surveillance may be useful for estimating community HSV-2 prevalence.

Herpes simplex virus type 2 (HSV-2) may both increase susceptibility to HIV infection and enhance its transmission. HSV-2 infection is associated with a threefold increased risk of HIV acquisition in both men and women.^1^ ^2^ HSV-2 prevalence is high in many populations, especially in Africa;^3^ it is possible that HSV-2 is a major determinant of the differential distribution of HIV between populations.^4^ ^5^ It is, however, currently unclear whether the high rates of HSV-2 now seen in association with HIV are a cause or, to some extent, a consequence of the high prevalence of HIV. HIV co-infection has been associated with increased severity and duration of HSV-2 ulceration and increased HSV-2 shedding,^6^ so increased transmission of HSV-2 is likely. There is no information available on long-term trends of HSV-2 in Africa.

In Karonga district, Malawi, we assessed HSV-2 prevalence over the period in which HIV prevalence rose from 4% to 12%.^7^ We also assessed socioeconomic determinants of HSV-2, associations with HIV in different time periods and the possible use of antenatal clinic (ANC) surveillance for measuring HSV-2 prevalence.

## METHODS

Karonga district is a rural area in northern Malawi with a population of approximately 250 000. Total population surveys were conducted in Karonga district in 1981–4 and 1986–9 for studies of mycobacterial disease. A case–control study of tuberculosis and leprosy was started in 1988; controls were selected from people seen in the second survey, age, sex and area matched to the cases, with up to four controls per case^8^ ^9^ Individuals who had moved away and any new immigrants were not included.

A new case–control study of tuberculosis was started in 1998. Controls were stratum-matched to the cases by age, sex and area, each year. They were selected using field-based random sampling.^10^ Random starting points in the field were chosen in proportion to population density in the second survey; a direction was chosen using a spinning top and the first person within a pre-specified age and sex band was selected. The controls at each period were therefore a random sample of the general population, but with an age, sex and area distribution similar to that of tuberculosis and leprosy cases. From 1998 new immigrants were included. Only the controls from these case–control studies were used in these analyses.

Controls were interviewed and blood was taken for HIV testing, after counselling and if consent was given. For controls seen in 1988–90 information on socioeconomic variables was only available from the 1981–4 survey and was not used for this analysis. Socioeconomic data were collected during the 1998–2005 household visits. ANC surveillance for HIV was started in 1999 in four clinics using unlinked anonymous testing.^11^

In 1988–90 initial HIV testing used an ELISA (Vironostika; Organon Teknika, Cambridge, UK) and particle agglutination test (Edgeware modification of the Serodia). Positives were confirmed using a further ELISA (Wellcozyme Wellcome Diagnostics, Dartford, UK or Enzygnost, Behring, Marburg, Germany) and particle agglutination assay (Serodia, Fujirebio Inc, Tokyo, Japan). From 1998, the first two tests were conducted as before, and samples giving discrepant results were repeated in duplicate using the same two tests. Western blot and repeat testing was used if there were continued discrepancies. From 1998 to 2001 individuals who did not want to give serum samples could give urine samples and from 2002 they could give saliva samples. Urine testing used GACPAT;^12^ results that were not clearly negative on the first test were repeated and any doubtful results were excluded. Saliva testing initially used GACELISA for a few early samples and Orasure Advance (Orasure Technologies Inc, Bethlehem, Pennsylvania, USA) rapid tests in the laboratory for the majority.

Stored sera from the controls and the ANC surveillance were tested for HSV-2 using a type-2-specific enzyme immunoassay (Kalon Biological Ltd, Surrey, UK), which has the highest sensitivity and specificity of commercially available assays when used on African samples.^13^ Sera from people aged 15–44 years were tested in 1988–90 and 2002–5, and those from all adults aged 15 years and over from 1998 to 2001. Permission for the original studies, and testing of the stored sera for HSV-2, was received from the Health Sciences Research Committee, Malawi, and from the ethics committee of the London School of Hygiene and Tropical Medicine.

Analyses compared the prevalence of HSV-2 at different periods, allowing for age, sex and area. Prevalence was estimated using direct standardisation, taking the population of the district in 1998 as the standard, using 5-year age groups and six areas.^11^ Because of the different methods of control selection, prevalence was also re-calculated for the later periods excluding those who had moved in the past 5 years, to make them comparable with the controls from 1988–90. Sociodemographic determinants of HSV-2 were assessed using logistic regression, using age, sex and area as a priori confounders. The association between HSV-2 and HIV seropositivity was explored.

HSV-2 prevalence in women attending ANC in 1999–2000 was compared with that in women in the community in 1998–2001. Factors associated with differences in prevalence were explored in a logistic regression analysis by assessing which factors confounded the association between ANC use and HSV-2 status.^14^ All analyses used STATA version 9.

## RESULTS

HSV-2 results were available from 677 controls from 1988 to 1990, 719 from 1998 to 2001, 383 from 2002 to 2005 and from 981 women attending ANC.

In the early period, 8.6% of controls refused venepuncture. HSV-2 testing was limited to adults aged 15–44 years. Of 811 specimens, a consecutive series of 116 were lost from storage. Of the remaining 695 sera, 14 were missing and four gave equivocal results for HSV-2.

In 1998–2001, of 995 individuals seen as controls, 728 (73%) gave blood for HIV testing (62% of the remainder gave urine specimens and the rest refused testing). Of the 728 specimens, two gave equivocal results for HSV-2 and seven were missing. Among those without HSV-2 results available in this period, women, the youngest and oldest age groups, those living in the urban and periurban area and those without schooling were overrepresented. The other sociodemographic factors and HIV prevalence were similar in those with and without HSV-2 results.

In 2002–5 saliva testing was used rather than urine for those not wanting to give blood for HIV. Of 573 controls aged 15–44 years, 424 (74%) gave blood for testing and 69% of the rest gave saliva. Of the 424, HSV-2 results were equivocal for three and missing for 38. In this period, women, younger and older age groups, those living in the southern and central parts of the district and those who were HIV positive were overrepresented among those without HSV-2 results.

### HSV-2 prevalence

HSV-2 prevalence by age, sex and time period, including directly standardised estimates, is shown in table 1. HSV-2 prevalence rose with age in all periods and was higher in women than in men, reaching 70% by age 40 years. The difference in HSV-2 prevalence between periods was most marked in the younger age groups but was similar for men and women. Restricting to those aged 15–44 years, HSV-2 prevalence was higher in 1998–2001 than in 1988–90: odds ratio (OR) 1.47, 95% CI 1.14 to 1.90 after adjusting for age, sex and area, but similar in the latest period (adjusted OR 1.15, 95% CI 0.86 to 1.52). After excluding those who had moved within the past 5 years from the two later periods, to make the control selection more equivalent to that in the earliest period, the age–sex–area-adjusted OR was 1.24 (0.93 to 1.66) in 1998–2001 and 1.11 (0.80 to 1.53) in 2002–5. Among those aged less than 25 years, excluding those who had moved in the past 5 years, HSV-2 prevalence was 24% (52/216) in 1988–90 and 22% (15/68) in 1998–2005 (combined due to small numbers); age–sex–area-adjusted OR 0.73 (0.35 to 1.53).

**Table 1:** HSV-2 prevalence in different time periods

	1988–90				1998–2001				2002–5				1999–2000	
	Men		Women		Men		Women		Men		Women		ANC women	
	HSV-2+/N	%	HSV-2+/N	%	HSV-2+/N	%	HSV-2+/N	%	HSV-2+/N	%	HSV-2+/N	%	HSV-2+/N	%
Total	111/334	33.2	192/343	56.0	191/335	57.0	256/384	66.7	75/178	42.1	114/205	55.6	467/981†	47.6
Age group, years														
15–19	12/75	16.0	13/44	29.6	2/13	15.4	8/20	40.0	0/13	0.0	1/20	5.0	66/246	26.8
20–24	10/54	18.5	17/43	39.5	4/15	26.7	28/50	56.0	3/18	16.7	12/27	44.4	136/316	43.0
25–29	22/52	42.3	30/55	54.6	11/24	45.8	33/54	61.1	12/43	27.9	35/66	53.0	124/208	59.6
30–34	17/48	35.4	40/67	59.7	27/55	49.1	52/75	69.3	21/39	53.9	28/45	62.2	84/118	71.2
35–39	25/52	48.1	46/70	65.7	32/52	61.5	42/57	73.7	22/33	66.7	17/23	73.9	46/72	63.9
40–44	25/53	47.2	46/64	71.9	22/41	53.7	39/50	78.0	17/32	53.1	21/24	87.5	9/17	52.9
45–49					34/44	77.3	18/25	72.0						
50–54					11/22	50.0	16/20	80.0						
55+					48/69	69.6	20/33	60.6						
Area (age 15–44 years)														
1 (rural + truck stop)	25/74	33.8	34/68	50.0	18/41	43.9	36/55	65.5	9/32	28.1	13/27	48.2	94/205	45.9
2 (rural)	45/104	43.3	34/70	48.6	26/40	65.0	32/52	61.5	15/32	46.9	16/24	66.7	112/239	46.9
3 (peri-urban)	8/41	19.5	39/76	51.3	14/33	42.4	32/54	59.3	15/37	40.5	23/37	62.2	46/102	45.1
4 (urban)	7/16	43.8	5/13	38.5	19/36	52.8	46/67	68.7	18/42	42.9	25/47	53.2	74/147	50.3
5 (rural + trading area)	9/25	36.0	32/47	68.1	12/27	44.4	32/46	69.6	11/17	64.7	22/41	53.7	101/208	48.6
6 (rural + border)	17/72	23.6	45/65	69.2	9/23	39.1	24/32	75.0	6/15	40.0	13/23	56.5	36/74	48.7
Standardised* HSV-2 prevalence age 15–44% (95% CI)														
Age-adjusted	29.6 (24.8 to 34.4)		47.5 (41.6 to 53.4)		35.6 (27.0 to 44.2)		58.0 (50.7 to 65.3)		27.0 (21.7 to 32.4)		44.3 (38.0 to 50.5)		48.2 (44.7 to 51.6)	
Age–area-adjusted	26.0 (20.9 to 31.0)		45.9 (40.8 to 51.0)		34.7 (28.7 to 40.8)		58.0 (52.2 to 63.8)		28.9 (24.4 to 33.3)		45.4 (40.3 to 50.4)		47.2 (44.1 to 50.3)	
Age-adjusted, excluding movers	29.6 (24.8 to 34.4)		47.5 (41.6 to 53.4)		27.7 (22.2 to 33.3)		55.4 (42.5 to 68.4)		25.6 (20.6 to 30.6)		37.0 (28.9 to 45.1)		47.8 (43.3 to 52.2)	
Standardised* HIV prevalence age 15–44% (95% CI)														
Age–area-adjusted	4.0 (2.0 to 5.9)		4.3 (2.5 to 6.1)		11.6 (7.2 to 15.9)		14.4 (10.5 to 18.4)		12.3 (8.1 to 16.5)		16.3 (12.0 to 20.5)			

*Directly standardised to the age and area distribution of the district. †Two aged <15 years, one aged >44 years and one with missing age excluded from further analyses.

ANC, antenatal clinic; HSV-2, herpes simplex virus type 2.

### Associations with socioeconomic status, 1998–2005

Information on socioeconomic factors was only available for the years 1998–2005 (table 2). After adjusting for age, sex, area and time period, the HSV-2 prevalence was similar in farmers and non-farmers, and varied little by schooling level or housing type. It was higher in those who had moved in the past 5 years and those who had most household possessions. The associations with movement and possessions became slightly stronger after additionally adjusting for each other.

**Table 2:** Associations between socioeconomic factors and HSV-2, 1998–2005, in adults aged 15–44 years

	Men		Women		Crude OR	OR adjusted for age, sex, area, and period OR (95% CI)	OR adjusted for age, sex, area, period and moved in past 5 years or possession score OR (95% CI)
	HSV-2+/N	%	HSV-2+/N	%	OR (95% CI)		
Moved in past 5 years					p = 0.2	p<0.001	p<0.001
No move	136/271	50.2	180/307	58.6	1	1	1
From elsewhere in district	12/62	19.4	96/146	65.8	0.90 (0.6 to 1.23)	1.40 (0.96 to 2.05)	1.50 (1.01 to 2.23)
From outside district	25/45	55.6	40/58	69.0	1.4 (0.92 to 2.18)	2.60 (1.57 to 4.28)	2.64 (1.59 to 4.40)
Occupation					p = 0.4	p = 0.6	
Farmers	96/222	43.2	219/343	63.9	1	1	
Non-farmers	71/150	47.3	93/162	57.4	0.88 (0.67 to 1.16)	1.09 (0.78 to 1.52)	
Schooling					p = 0.002	p = 0.4	
<6 Years primary	28/56	50.0	132/197	67.0	1	1	
6–8 Years primary	86/184	46.7	139/236	58.9	0.67 (0.49 to 0.92)	0.79 (0.55 to 1.12)	
Secondary/tertiary	58/137	42.3	44/76	57.9	0.53 (0.37 to 0.77)	0.87 (0.56 to 1.34)	
Housing score					p = 0.9	p = 0.7	
1 (worst)	48/117	41.0	107/166	64.5	1	1	
2	32/64	50.0	42/77	54.6	0.91 (0.61 to 1.37)	0.92 (0.59 to 1.44)	
3	60/120	50.0	96/163	58.9	1.01 (0.73 to 1.41)	1.11 (0.76 to 1.62)	
4 (best)	29/71	40.9	65/96	67.7	1.06 (0.72 to 1.56)	1.22 (0.77 to 1.93)	
Possession score					p = 0.004	p = 0.02	p = 0.03
1 (worst)	20/50	40.0	41/65	63.1	1	1	1
2	39/95	41.1	81/136	59.6	0.96 (0.61 to 1.50)	1.0 (0.61 to 1.64)	1.02 (0.62 to 1.67)
3	81/171	47.4	115/208	55.3	0.95 (0.62 to 1.44)	0.92 (0.58 to 1.45)	0.98 (0.61 to 1.56)
4 (best)	31/57	54.4	73/96	76.0	1.88 (1.14 to 3.10)	1.79 (1.03 to 3.10)	1.86 (1.06 to 3.25)

Housing score depends on housing materials.

Possession score depends on the number of household items (bicycle, motor vehicle, canoe/oxcart, bankbook, clock, radio, cattle) owned, weighted by relative value.

HSV-2, herpes simplex virus type 2; OR, odds ratio.

### Associations with HIV status in adults aged 15–44 years

In 1988–90 1.4% (3/222) HSV-2-negative men and 6.4% (7/110) HSV-2-positive men were HIV positive. For women the results were 2.0% (3/151) and 5.7% (11/192). The OR for the association of HSV-2 and HIV, adjusted for age and area, was 4.0 (0.97 to 17) for men and 3.1 (0.78 to 12.2) for women. In 1998–2005, among men, 4.4% (9/204) of HSV-2 negatives and 27% (47/173) of HSV-2 positives were HIV positive, OR, adjusted for age, area and time period, 7.8 (3.5 to 7.4). Among women equivalent figures were 4.7% (9/192), 26.0% (82/316) and 9.0 (4.1–19.7).

### HSV-2 prevalence in ANC (1999–2000) and community (1998–2001)

HSV-2 prevalence was lower in women in the ANC group than in the community in the same period (table 1). Among women aged 15–44 years, those in the ANC group were younger than those in the community (mean 24.5 years compared with 30.9), came from slightly different areas, were more likely to be currently married (93% compared with 79%), were less likely to have previous children, were more likely to have moved recently within the district, but were less likely to have moved from outside and had a higher level of schooling. Similar proportions came from farming households. Comparing the crude prevalence of HSV-2 between ANC women and those in the community, the OR was 0.47 (0.36 to 0.61). This was largely explained by the age difference: age-adjusted OR 0.72 (0.54 to 0.97). Additionally adjusting for area made no difference. Additionally adjusting for marital status increased the OR to 0.79 (0.58 to 1.07); adding movement in the past 5 years increased the OR to 0.83 (0.61 to 1.13) and adding previous children increased it further to 0.85 (0.62 to 1.7). Adjusting for schooling and occupation made no difference to the results. (Housing and possession information was not available in the ANC.) The age and area-standardised prevalence in the ANC group (47.2%, table 1) was similar to that for men and women combined (46.4%).

**Key messages**

- Although HIV infection increases HSV-2 transmission, we show that HSV-2 prevalence was already high in the early years of the HIV epidemic and was not greatly influenced by increasing HIV prevalence.
- ANC surveillance can be used to estimate population level HSV-2 prevalence.

## DISCUSSION

HSV-2 prevalence was already high in the late 1980s, when HIV prevalence was low, so the high prevalence is not a result of the HIV epidemic. An apparent increase in HSV-2 prevalence in the late 1990s is largely explicable by the inclusion of individuals who had moved recently. In all time periods the community samples were randomly selected, and after adjusting for age, sex and area, should have been representative of the whole district. In the early period refusal rates were low and the lost sera will not have biased the results, but individuals who had moved since the survey were not included. In the later surveys movement was associated with higher HSV-2 prevalence. After excluding those who had moved from the later surveys, the difference between the periods was much reduced and not significant. A higher HSV-2 prevalence in those who are more mobile is not unexpected, as this is often found for HIV, reflecting higher sexual risk and movement related to marriage.^15^

In the later surveys refusal rates were higher and there were some differences between those who refused and those who did not. Differences in age, sex and area were accounted for in the analysis, but the underrepresentation of those with no schooling among those tested may have led to a slight overestimate of HSV-2 prevalence. On the other hand, HIV prevalence was higher in those who gave saliva samples; there may have been some false positives, or high-risk individuals may have refused blood tests, which would underestimate HSV-2 prevalence in the most recent period.

The data therefore provide little evidence of a real increase in HSV-2 prevalence over time. We have previously shown that HIV rose earliest and highest in the urban area,^16^ but this is not reflected in the HSV-2 prevalence distribution, consistent with HIV not having a major influence on HSV-2 prevalence. In rural Uganda and Tanzania, HSV-2 prevalence was also already high in the general population by 1989/1992,^3^ and high HSV-2 prevalence has been reported in West Africa in the 1980s, although the age and population groups are not clear.^17^ This suggests that HSV-2 was already widespread in Africa before the HIV epidemic, and that HIV infection is not an important determinant of the HSV-2 prevalence in the population. This conclusion is also supported by mathematical modelling of the Ugandan HIV epidemic, in which stable HSV-2 prevalence was attributed to the small increase due to enhanced transmission by HIV being balanced by the effects of HIV-related mortality,^18^ and by modelling of the interaction of HIV and HSV-2 in Kisumu, Kenya.^19^

ANC data are used in HIV surveillance, and the biases are well known.^20^ Here we explored the potential of ANC data for HSV-2 prevalence estimation. As for HIV, the ANC data underestimated prevalence. This was seen in all age groups, whereas an overestimation in the youngest age group would be expected, reflecting differences in sexual activity; however, the number of young women among the community controls was small. For HIV, one bias is the direct biological effect on fertility, whereas for HSV-2 the bias appears to reflect sociodemographic differences in fertility and hence ANC use. As has previously been shown for HIV, the lower estimate of prevalence for women from the ANC group compared with the population, together with the higher prevalence in women than in men in the population, result in the prevalence of HSV-2 in the ANC group approximating to that in the population for men and women combined.

HSV-2 infection is a major determinant of HIV infection risk at the individual and perhaps at the population level.^5^ We provide further evidence that HSV-2 was widespread in Africa before HIV and is not simply a result of the HIV epidemic, and suggest that HSV-2 prevalence can be estimated using ANC surveillance.
